# Microscopical Appearances in the Intestinal Discharges and Muscular Fibres of a Patient Who Died of Epidemic Cholera

**Published:** 1849-08

**Authors:** 


*Microscopical appearances in the Intestinal discharges and muscular fibres of a patient who died of epidemic cholera.*—Dr. Waldo Burnett, in a paper reap at a meeting of the Boston Society for Medical Improvement, (for an abstract of which see American Journal of the Medical Sciences,

No. for July, 1849,) stated the results of repeated microscopical examinations of the "rice water" liquid found in the intestines of a patient dead with epidemic cholera. It was found to oder with epithelium, *cylinder* and *tesselate.* The latter variety of epithelium covers the mucous membrane of the mouth and œsophagus, while the former variety covers the remaining portion of the alimentary canal. Hence, the presence of both varieties, shows that the discharges come from the whole of the alimentary canal, and not from the intestinal portion alone. Crystals of the *oxalate of lime* were also observed. But the most interesting of the appearances were, what seemed to Dr. Burnett, and also to Dr. D. H. Storer, (who was present,) an infinite number of animalculæ "swarming and sporting about in every direction." They appeared to belong to the infusory genus, *vibriones.* On submitting a portion of the pectoral muscle to examination by the microscope, the same animalculæ were found in great numbers.

These results are, to say the least, curious, and should excite to further investigations.

